# Syphilis in Its Relation to Dental and Oral Surgery

**Published:** 1886-08

**Authors:** G. Frank Lydston


### Syphilis in Its Relations to Dental and Oral Surgery.

BY G. FRANK LYDSTON, M.D.,

PROFESSOR OF SURGERY IN THE NORTHWESTERN COLLEGE OF DENTAL SURGERY; LECTURER ON GENITO-URINARY AND VENEREAL DISEASES IN THE COLLEGE OF PHYSICIANS AND SURGEONS, CHICAGO, ILLINOIS.

(Read in the Section of Dental and Oral Surgery, on May 4th, at the Thirty-Seventh Annual Meeting of the American Medical Association.)

The time is long since past when an apology was necessary for bringing a subject of general surgery before a convention of dental and oral surgeons. Dental and oral surgery has, of late years, come to be recognized as a specialty of general surgery, and most justly so. The progressive dentist of to-day is earnestly striving to attain all of the qualifications necessary to the true specialist, who has been so aptly described as a practitioner "who knows something of everything, and everything of something." There is perhaps no specialty that involves more of the principles of general medicine than that of dental surgery. It is greatly to the credit of the dental profession that it is now recognizing this fact.

There is perhaps no general disease which figures as a more important factor in dental and oral practice than syphilis. The prevalence of this disease, as revealed to the general surgeons and specialists of our large cities, is somewhat appalling, and when we stop to consider its insidiousness, the importance of the subject is at once appreciated. The oral symptoms of the disease, in well-managed cases, are now-a-days the most important of all the local manifestations which characterize its active period. The reasons for this are at once apparent. The vast majority of well-treated cases will escape skin lesions altogether, or at most will have nothing more severe than the roseola, which is of the most trifling importance, but very few indeed will be found to go through a course of syphilis without being afflicted with oral symptoms of greater or less severity. Such, at least, has been my experience in quite an extensive field of observation. The argument may be advanced that this statement applies only to the better class of patients; but it is just this class whom the dentist is most frequently called upon to treat. When the facts seem to controvert the proposition which has been advanced, they seem to be the result of causes independent of the intrinsic severity of syphilis. Another consideration that tends to enhance the importance of the oral syphilides, is the fact that it is through these lesions that innocent persons are most apt to become infected.

In the matter of prophylaxis of oral lesions in syphilis, the function of the dentist is acknowledged by progressive specialists, to be of paramount importance. The patient who would go through a course of syphilis and of treatment therefor, with a minimum of suffering, will place himself in the hands of his dentist, as preparatory to undergoing strictly medicinal treatment. The patient with tartar about the teeth, carious cavities, or sharp or rough surfaces upon the teeth, jutting against the buccal mucous membrane or tongue, is almost sure to suffer from luxuriant crops of mucous patches, or destructive ulcerations. In addition to this inconvenience, we will find that under the faulty conditions mentioned, reflex excitation of the salivary and mucous glands is constant, and when under such circumstances the stimulating effect of mercury is superadded, ptytalism or even stomatitis is quite likely to occur. With the teeth and gums in bad condition, the patient is usually so intolerant of mercury that he cannot take a sufficient quantity to benefit his syphilis.

In passing, it may be well to caution against carelessness in the matter of cleansing all instruments used in dental practice, whether the patient be known to be syphilitic or not. Syphilis is so widely disseminated, even among respectable people, that it is unsafe to run any risk in the matter. The oral lesions of syphilis are very actively contagious during the early months of the disease, and it has been supposed that they retain their contagious properties for a much more prolonged period than do other lesions. A lesion so insignificant as to escape attention, may give rise to a highly contagious secretion. This secretion becomes mingled with the saliva and may adhere to instruments for an indefinite time. Just how long the contagion may retain its vitality is unknown, but it is probably a prolonged period. I am happy to say that very few, if any, accidents have been laid at the door of the dentist, but that fact should not render him one whit less cautious. I have myself seen one case in which there were strong grounds for suspicion of infection by dental instruments. It is to be said, however, that the dentist in question was not a reputable member of the profession.

A certain preventive of such accidents is to dip all instruments in pure carbolic acid, both before and after using, in addition to the ordinary measures of cleanliness. Ordinary solutions of antiseptics are too weak to be efficacious. Iodine and bichloride of mercury corrode the instruments, and the flame of the alcohol lamp destroys their temper, hence pure carbolic acid is the most efficient and available antiseptic. If prepared in the manner suggested, infection from instruments is impossible. A safe rule in surgery is to avoid the use of an instrument which we would hesitate to put in our own mouths, or thrust in our own flesh, if necessary.

In beginning the discussion of syphilis as an etiological factor in dental and oral lesions, it is proper to consider the question of hereditary syphilis. This is a subject of much more importance to the dentist than is generally supposed. It has long been well understood that syphilis is capable of producing marked disturbances of the nutrition and development of the teeth. This fact has been developed chiefly through the efforts of the renowned syphilographer, Mr. Jonathan Hutchinson, of London. It may seem presumptious to criticise anything emanating from so eminent an authority, but it is my humble opinion that Mr. Hutchinson's description of the teeth and facies of the syphilitic child, has tended to mislead us. Observe his original description: "The permanent teeth are irregular and defective; they are small, vertically notched, rounded at the corners or pegged, and are often marked with seams and ridges or lines, of a dirty brownish color. These characters are especially marked in the central incisors, which are the test teeth." He further says: "Next in value to the malformation of the teeth, are the state of the patient's skin, the formation of his nose, and the contour of his forehead; the skin is almost always thick, pasty, and opaque. It also shows pits and scars, the relics of former eruptions, and at the angles of the mouth are radiating linear scars, running out into the cheeks. The bridge of the nose is almost always low, and broader than usual; often it is remarkably sunken and expanded. The forehead is usually large and protuberant in the region of the frontal eminences; and often there is a well-marked depression just above the eyebrows. The hair is usually dry and thin, and now and then the nails are broken and splitting into layers."

It would be interesting to know how many cases each of the gentlemen present have had under observation, that tallied with the above description. Exceptionally, indeed, has it been my privilege to observe such a *tout ensemble* of symptoms, yet it has been my lot to observe and treat many unequivocally syphilitic children. It has been the custom to look for Hutchinson's test teeth; seldom indeed are they found; ergo, the patient is not syphilitic.

Now, as a matter of fact, the children who are so badly affected with syphilis as those described by Hutchinson, seldom arrive at the period of dentition, for the vast majority of the victims of severe hereditary syphilis fail to survive the third month after birth. It is the child who suffers from attenuated hereditary syphilis who survives, and it is with him that the dentist has to deal; hence the extreme cases described by Hutchinson are not fair criteria of the effects of syphilis upon the teeth, as seen in dental and oral practice, although such cases may be met with.

When I use the term attenuated syphilis, I mean hereditary infection not severe enough to cause typical syphilitic lesions, but severe enough to cause malnutrition. Like all conditions of perverted nutrition occurring during the period of dentition, that produced by hereditary syphilis gives rise to defective and irregular development, faulty texture, and early decay of the teeth. The syphilitic cachexia is pre-eminently liable to produce such results, but unfortunately there is nothing characteristic in the resulting physical appearances, in the greater proportion of cases.

Very frequently (perhaps generally) if the constitutional element be recognized at all, it is dubbed "scrofula" or "rickets!" Old Proteus masquerading under a respectable name; but none the less susceptible to the eloquent persuasion of our reliable friends, mercury and iodine. I venture to say that in quite a proportion of troublesome cases of dental caries and irregularties in children, constitutional treatment of this kind will prove a very useful auxiliary to the efforts of the dentist.

In the consideration of this important question, there are several facts to be borne in mind by the dental surgeon. These may be formulated as follows: 1st. The great frequency of syphilis, even in refined society. 2d. The recklessness as to the consequences of matrimony exhibited by the average syphilitic patient. 3d. The great percentage of danger of hereditary transmission of even remote syphilic taint. 4th. The large number of tainted children that he must necessarily be called upon to treat as a consequence of the foregoing propositions. 5th. That Hutchinson's test teeth and facies syphilitica are by no means a *sine qua non* in hereditary syphilis. It may be readily observed that the duty of the dental surgeon is not completed with the local treatment of such cases as those under consideration, but he should recommend a suitable course of constitutional treatment. The time is coming when every dentist may not only recommend, but will be competent to carry out, such a course in detail.

The co-relation of so-called struma and hereditary syphilis has of late years come to be suspected, and the tendency in certain quarters to regard many cases of alleged scrofulous lesions as remote manifestations of syphilitic taint, is rapidly gaining ground. This, indeed, was tacitly admitted years ago by Astley Cooper, whose favorite remedy for scrofula consisted of the bichloride of mercury, in Huxham's tincture of cinchona bark.

In connection with the subject of hereditary syphilis, it is well to remember that there is such a thing as late hereditary syphilis, in which, while the patient escapes all manifestations of the disease during childhood, save perhaps malformed or crumbling teeth, lesions of the mouth, jaws, and nasal cavities appear later in life. Ziessl has collected over one hundred of such cases, and has reported a number observed by himself. These cases strongly resemble tertiary acquired syphilis, and appear, as a rule, about the period of adolescence. Osseous and nervous lesions of a severe character usually predominate, the osseous lesions having a special predilection for the nasal and palatal bones. I recall a case of caries of the skull of this character.

It may be remarked in passing that many cases of the ozæna, or fetid nasal catarrh, with resulting necrosis or caries of bone or cartilage, are attributed to simple catarrhal inflammation, the diagnosis being based mainly upon the absence of specific history or typical lesions. Most of the cases are the result of acquired syphilis, but some of them undoubtedly are late manifestations of hereditary taint. As far as my personal opinion is concerned, I have been unable to convince myself that simple catarrh is ever the cause of nasal or palatal necrosis or caries. I recall two cases in which failure to accept this view on the part of the family physician, resulted in serious and irreparable disfigurement. To apply this to dental and oral practice, it is only necessary to remember that necrosis or caries of the palate or jaws, accompanied by or dependendent upon, alleged fetid nasal catarrh or ozæna, can, with almost absolute safety, be pronounced syphilitic. It must be borne in mind, however, that such lesions may represent the errors or misfortunes of a past generation, and may therefore cast no discredit upon the respectability of the patient.

The primary lesion of acquired syphilis very rarely occurs within the mouth; chancre of the lip being more frequent. The dental surgeon will therefore seldom meet with it. I have already alluded to a case in which infection by dental instruments was the presumable origin of the disease. Unfortunately, I did not have an opportunity to observe the case from its inception. At the time I first examined the patient, secondary sore throat and mucous patches had already appeared. A small sluggish indurated ulcer existed upon the gums, below the lower middle incisors. The glands beneath the jaw were the first and only glandular changes to attract the attention of the patient, and they were disproportionally enlarged as compared with the rest of the glandular system. It was claimed by the patient that the sore upon the gums appeared about three weeks after having had her teeth cleaned and put in order. The "kernels," as she termed them, beneath the jaw, appeared about a week or ten days later. There were no evidences of venereal infection, and as far as physical appearances went she had never been exposed to venereal contagion.

I recall another case of interest in which the primary lesion appeared upon the tip of the tongue. There was very little induration about the sore, which appeared as a shallow ulcer the size of a split pea, with a grayish base covered with an exudate very like a diphtheritic membrane. The glands under the jaw enlarged, and the case ran the ordinary course of syphilis. The source of infection was traced, and I found mucous patches in the mouth of the woman from whom the disease was contracted. She herself gave a clear history of having contracted her syphilis from her husband. In this case, a diagnosis could have been made in no other way than by tracing the source of infection, and by observation of the subsequent course of the case, inasmuch as the primary lesion was decided atypical.

The oral symptoms of the secondary or active period of acquired syphilis are of the most vital importance to the dental surgeon, by virtue of 1st, their contagiousness; 2d, their liability to be mistaken for innocuous lesions; 3d, the prolonged period during which they are apt to develop at any time, and often without the patient's knowledge; 4th, the necessity for nearly all syphilitics to consult their dentist sooner or later, during the active period of syphilis. The later, or so-called tertiary lesions, are so destructive that they usually announce their presence at once; and more important still, they are probably rarely contagious.

The lesions of the mouth occurring during the course of constitutional syphilis may be briefly classified as: 1st. Excoriations or erosions, and rhagades, or fissures, occurring with or without more or less generalized inflammation of mucous membrane. 2d. Mucous patches and tubercles. 3d. Early ulcerations, superficial or deep. 4th. Late ulcerations, superficial or deep. 5th. Early and late bone and periosteal lesions.

Excoriations or erosions and fissures are the most dangerous to the safety of healthy persons, from the fact that they are often precisely identical in their physical characters with similar lesions from simple causes. Their secretion, however, is extremely contagious, and the act of kissing may be followed by disastrous results. Fortunately, these lesions are usually quite irritable, occurring, as they generally do, in smokers, and they consequently usually announce their presence most emphatically. These lesions may appear at any time during the course of syphilis, but are most often seen during the first year. They are due to the co-existence of local irritation with constitutional depravity, and as both of these conditions occur from simple causes, it is obvious that great caution is necessary in the matter of diagnosis.

These erosions and fissures are distinguished in most cases by the generally reddened and tender state of the mucous membrane. Unlike other syphilitic lesions, they are sometimes extremely painful, the suffering being greatly disproportionate to the actual lesion present. A diagnosis of syphilis is not warranted in these lesions unless confirmed by more positive indications than those afforded by the lesions themselves.

The typical mucous patch is the most frequent of the oral lesions of syphilis. It represents a papule upon a mucous surface, and also represents the foundation of most of the ulcerative lesions of the disease, *i. e.*, a localized proliferation of cells. It is a true hyperplasia of tissue, and this is an important element in the diagnosis. The patch is elevated, of a grayish or yellowish color, and varies in size from a scarcely perceptible point to a patch or collection of patches the size of a silver quarter, rarely larger. There is no inflammatory area about it as a rule, but if irritated, the ordinary characters of inflamed mucous membrane will be presented.

There is a peculiar form of mucous patch which is sometimes seen about the borders of the tongue, that merits special description. It is the most obstinate of the lesions of the tongue, excepting, perhaps, the rare form known as syphilitic psoriasis of the tongue. It appears in the form of a furrow or cleft in the border of the organ upon one or both sides. This furrow has an appearance resembling a puckering in or involution of the mucous membrane. The borders of the lesion are rounded, and covered with a fibrous layer of cloudy epithelium, which looks very much like a thin film of coagulated albumen.

The mucous tubercle is an exaggerated mucous patch, and consists of a similar but more extensive aggregation of cells. Either the patch or the tubercle may go on to ulceration.

There is a peculiar tendency in some cases of mucous patches to a circinate arrangement very like ordinary ringworm of the skin in form. This is due to a coalescence of circular patches, and is especially marked upon the roof of the mouth, involving both the hard palate and velum. When this circinate eruption is seen in the mouth, nothing more is wanted to complete the diagnosis of syphilis, although corroborative evidence should always be sought for.

The special points which are to be sought for in the diagnosis of syphilis will serve in all cases, save in late syphilis, where we may be compelled to depend upon the history and the appearance of the lesions alone.

These evidences are: 1st. A diffuse passive congestion or hyperæmia of the fauces and pharynx, without corresponding pain in deglutition. Should the patient have a bad cold at the time, this sign ceases to be of great value. 2d. Alopecia or falling of the hair, in the absence of parasitic disease. 3. Coppery spots or cicatrices; the remnants of faded skin lesions. 4. The presence of scabby spots or pimples on the scalp. 5th. Enlargement of the lymphatic glands, those of the neck being especially accessible. Enlargement of the sub-occipital glands is pathognomonic. 6th. A possible history of bone pains and aches, worse at night; this sign being more important in late syphilis. 7. The presence of circumscribed periosteal swellings or nodes.

It will be observed that most of these signs can be sought for and often found by the dentist, without examination of any other regions than the oral cavity, pharynx, head, and neck. Indeed, a quite thorough examination may often be made, without exciting suspicion, if such a course be politic. For obvious reasons, I do not include some diagnostic points for which the general surgeon would seek. An innocent and to all appearances quite casual search for evidences of diseases as scrofula, rheumatism, and catarrh may develop some startling and valuable results in dental and oral practice. When the patient is sufficiently cosmopolitan or candid, the history of infection will clear up any possible obscurity. A useful point in the diagnosis is that the patient is usually distressingly healthy aside from the local manifestations of disease, and even these may be treated with great indifference.

There is a peculiar lesion of the tongue to which allusion has already been made, that merits special attention, although it must be confessed it is a lesion that is rarely seen. I refer to syphilitic lingual "psoriasis." It closely resembles simple psoriasis of the tongue, and is apt to make its appearance late in the course of the disease, when nothing but the history of the case remains to assist in the diagnosis. It consists of a thin whittish pellicle, due to thickened epithelium, the mucous membrane beneath having a peculiar scalded appearance. Quite often, the tongue presents large irregular patches of scalded looking mucous membrane. The borders of the tongue are most apt to be affected. That these lesions are dangerous is shown by a case reported by Fournier, in which a patient with this lesion infected a healthy woman by kissing, five years after he contracted the disease. It is the most obstinate of lesions, and will persist after all other evidences of syphilis have yielded to treatment. I have a case at the present time that is absolutely intractable to therapeutic measures.

There is a form of ulceration of the mucous membrane which occurs in debilitated states of the system, and is popularly known as "canker." It is met with especial frequency in nursing women. This is important, in view of the fact that it may be mistaken for syphilis, and *vice versa.* These erosions or ulcerations are superficial in character, usually multiple, and appear in successive crops. They are very difficult of cure sometimes, and require a treatment which is the direct antithesis of that required for syphilis. The eruption is probably a herpes, the basis of which is a neurosis, and in its treatment nervine tonics are indicated.

These lesions are preceded by simple hyperæmia, with little or no swelling and evidently no new formation. Their color is usually pearly white, or, if greatly inflamed, they present a yellowish base. They look very much as if the mucous membrane had been snipped out with the scissors. They are often quite painful. There are no evidences of constitutional taint in the form of glandular engorgement, excepting in some strumous cases, in whom a diagnosis may be entirely dependent upon the crucial test of treatment. Under mercury and iodine, these simple cases will get worse. A complete change of air and scene may be necessary for their cure. It is not well to be hasty in the matter of diagnosis, but it is indeed a safe rule that, to the surgeon, no one should be above suspicion.

Some of the very late lesions in the mouth in syphilitic subjects are very difficult of diagnosis, the history alone being all that we can depend upon. These lesions are slight erosions and exudations, and, as a rule, are not contagious. Oftentimes, perhaps, they are not really syphilitic, and depend upon, first, exhaustion and malnutrition produced by the previous general infection ; second, mercury in too free doses. Most often, both of these causes are contributing factors. It should be remembered that because a patient has once had syphilis is no reason that he should not thereafter have simple and respectable lesions of the oral cavity. The excessive use of mercury, or even its moderate use, in special cases of idiosyncrasy, may produce lesions of the greatest importance to the oral surgeon. Much uncalled for abuse has been heaped upon this valuable drug, but after all, it is sometimes responsible for morbid changes, especially in the mouth. It is debilitating in large doses, and as eliminated by the salivary glands it is apt to be irritating. I see many cases of supposed mucous patches and syphilitic ulcerations which I am inclined to attribute to mercury. When, therefore, I find that the mouth not only does not improve, but really grows worse, under mercury, I infer that the trouble is really due to the drug, and substitute iron and tonics. The horribly fetid odor of the breath, due to decomposing fat, and the sponginess and tenderness of the gums, will indicate the probable cause in these cases, for lesions due to syphilis *per se* rarely fail to improve under mercury, when carried to its full physiological effects. It is certainly a suggestive fact that mercury is capable of preventing skin lesions in a large proportion of cases of syphilis; but does not seem to completely prevent oral manifestations in the majority of cases. While not yet prepared to formulate a rule, I am free to say, that the patients whose mouths have given me the most trouble, have been those to whom mercury has been given most freely.

It has been noted by dental surgeons that mercury is capable of inducing the condition known as pyorrhœa alveolaris. From personal observation I am convinced of the correctness of this view. There is also little doubt in my mind as to the probability of syphilis itself being the cause in some cases. Although not familiar with dental literature upon this subject, I have found in conversation with several members of the dental profession, that cases occasionally occur which are attributed to syphilis. It is obvious that in these cases, constitutional treatment is of the greatest importance, and is the only method of accomplishing that basic principle of all treatment—removal of the cause.

Although the ordinary erosions, mucous patches, and ulcerations of the oral cavity occurring during the secondary period, are annoying, they are rarely destructive. This cannot be said of the late secondary and tertiary lesions. A characteristic feature of late syphilis is a tendency to the deposit of so-called gummy material or syphiloma in various situations. This consists essentially of an accumulation of cells of a low grade, which obstruct the tissues, and impair their vitality.

This deposit has a special predilection for the osseous tissues and periosteum, and often affects the jaws and hard palate, with or without involvement of the nasal bones. Its results are inflammation, caries, or necrosis. In the diagnosis we rely upon the history somewhat, but there is usually no difficulty in passing an opinion upon the physical appearances alone. If seen early, a gummy tumor may be detected. It is a hard inelastic swelling over the bone. It is not especially tender, and is not much discolored, as a rule, although it may be of a bluish tint. It is not very painful, except at night, when the pain is apt to be severe. Taken all in all, the morbid process is a passive one. Later on, the gumma softens at its centre, and the skin becomes thinned and discolored. Ulceration may occur, or under treatment the softened material may resolve. If the tumor absorbs, a depression remains in the surface of the bone. If ulceration occurs, the probe will usually strike bare and roughened bone at the bottom of the cavity. When small bones, such as the vomer and the palate, are involved, nutrition is so impaired, that necrosis occurs in many cases. In such cases the bone is apt to come away entire and preserve its natural form. Nothing but syphilis will thus dissect out a bone. The resulting deformity in these cases is very marked, and should the palate be involved, speech is so impaired that the aid of the dentist is necessary. With an obturator, these cases of acquired cleft palate may be made quite comfortable. Operation is rarely productive of great benefit on account of the low vitality of the tissues in long standing cases of syphilis.

It is probable that, with the exception of phosphorus necrosis, traumatic necrosis, and cases undoubtedly due to diseased condi- of the teeth, the majority of cases of necrosis of the jaws, nose, and palate are due to syphilis, more or less remote. The dental engine has done excellent service in these cases. In the hands of Dr. Goodwillie, especially, it has done good work. It has been shown that by early operation the necrosis may be limited and recovery hastened.

I have already encroached upon the time of the Section to a considerable extent, yet I have not been able to do more than outline in a general way the more important points of my subject. I am well aware that the ground has not been thoroughly covered, but if points in the individual experiences of the members have been recalled to mind, the object of this paper will have been accomplished.—*Jour. Am. Med. Assn.*

